# Est10: A Novel Alkaline Esterase Isolated from Bovine Rumen Belonging to the New Family XV of Lipolytic Enzymes

**DOI:** 10.1371/journal.pone.0126651

**Published:** 2015-05-14

**Authors:** María Cecilia Rodríguez, Inés Loaces, Vanesa Amarelle, Daniella Senatore, Andrés Iriarte, Elena Fabiano, Francisco Noya

**Affiliations:** 1 Departamento de Bioquímica y Genómica Microbianas, Instituto de Investigaciones Biológicas “Clemente Estable”, Montevideo, Uruguay; 2 Laboratorio de Ecología Microbiana, Instituto de Investigaciones Biológicas “Clemente Estable”, Montevideo, Uruguay; 3 Departamento de Genómica, Instituto de Investigaciones Biológicas “Clemente Estable”, Montevideo, Uruguay; University of Waikato, NEW ZEALAND

## Abstract

A metagenomic fosmid library from bovine rumen was used to identify clones with lipolytic activity. One positive clone was isolated. The gene responsible for the observed phenotype was identified by *in vitro* transposon mutagenesis and sequencing and was named *est*10. The 367 amino acids sequence harbors a signal peptide, the conserved secondary structure arrangement of alpha/beta hydrolases, and a GHSQG pentapeptide which is characteristic of esterases and lipases. Homology based 3D-modelling confirmed the conserved spatial orientation of the serine in a nucleophilic elbow. By sequence comparison, Est10 is related to hydrolases that are grouped into the non-specific Pfam family DUF3089 and to other characterized esterases that were recently classified into the new family XV of lipolytic enzymes. Est10 was heterologously expressed in *Escherichia coli* as a His-tagged fusion protein, purified and biochemically characterized. Est10 showed maximum activity towards C4 aliphatic chains and undetectable activity towards C10 and longer chains which prompted its classification as an esterase. However, it was able to efficiently catalyze the hydrolysis of aryl esters such as methyl phenylacetate and phenyl acetate. The optimum pH of this enzyme is 9.0, which is uncommon for esterases, and it exhibits an optimal temperature at 40°C. The activity of Est10 was inhibited by metal ions, detergents, chelating agents and additives. We have characterized an alkaline esterase produced by a still unidentified bacterium belonging to a recently proposed new family of esterases.

## Introduction

Lipolytic enzymes, such as carboxylesterases (EC 3.1.1.1) and triacylglycerol lipases (EC 3.1.1.3), have been extensively used in the manufacturing and processing of detergents, foodstuffs, drugs, paper, textiles, leathers, and fine chemicals, demonstrating their versatility for biotechnological applications [1, 2]. Collectively, they are active over a broad range of substrates but, individually, they can be highly selective and even stereo-selective. In general, they do not require cofactors and are stable in various organic solvents [3–8]. Lipolytic enzymes belong to the α/β-hydrolase superfamily and contain a catalytic triad that usually consists of a nucleophilic serine in a GXSXG pentapeptide motif and an acidic residue (aspartic acid or glutamic acid) that is hydrogen bonded to a histidine residue [9, 10]. The pentapeptide motif is usually located between a β-strand and a α-helix, and assumes an extremely sharp turn called the nucleophilic elbow [11].

These enzymes were originally classified into only eight families (I-VIII) based on their amino acid sequences and biological properties [12]. Later, more families were added (IX to XVI) [7, 13–16]. Recently, a new classification was proposed by Lenfant et. al. in which all the α/β-hydrolases deposited in the ESTHER database were divided into 148 families and superfamilies subsequently grouped into blocks (C, H, L, and X) [17]. Some of these families are comprised mostly of members that contain “Domains of Unknown Function” or DUFs, which have not been characterized experimentally yet. More than 20% of all protein domains are annotated as DUFs in the Pfam database and about 2,700 DUFs are found in bacteria [18, 19]. One family of α/β-hydrolases is characterized by the domain DUF3089 which is shared by all its 74 protein members. This family has been recently included in the ESTHER database classification and named family XV [17]. The first member of this family, EstD2, was characterized in 2010 [7], and four other members, Est5S, EstGK1, EstZ3 and EstWSD, were characterized later on [15, 16, 20]. All of them are enzymes that display esterase activities. The remaining 69 protein members have not been characterized yet and DUF 3089 is still classified as a family of proteins with no known function.

The sheer volume of genomic information that is available has overwhelmed our ability to explore functions of individual genes using conventional direct genetics and molecular biology approaches. Similarly, sequence-based metagenomics has produced a wealth of information but also faced the annotation hurdle [21]. In contrast, functional metagenomics turns the problem around by first identifying specific functions present in a microbial population and then isolating the genes responsible for them [22, 23]. To date, numerous novel biocatalysts from various microbial habitats, such as lipases, esterases, cellulases, proteases, amylases, lacasses, were identified by functional metagenomic approaches [5, 7, 20, 24–26].

In this study, we describe the identification and biochemical characterization of Est10, a novel esterase isolated from a bovine rumen metagenomic DNA library. According to sequence analysis, Est10 is a member of the family XV. It is active on short-chain fatty acids esters and some aromatic esters. It is active at alkaline pH which makes it attractive for biotechnological applications.

## Materials and Methods

### Sample collection and processing

One hundred and fifty grams of fresh cow rumen digesta of a Holando bull (2 years old, 482 kg, pasture fed in southern Uruguay) was collected from a slaughterhouse. Immediately after collection, the rumen sample was kept on ice and processed in the same day. The DNA extraction was based on a modification of a method described previously [27]. The liquid fraction or Lq of digesta was obtained by compressing whole digesta between two layers of cheesecloth. The cells were harvested from this fraction by centrifugation at 10.000×g for 20 min at room temperature. The cells were suspended in 1 ml of PBS buffer pH 8.0.

### Isolation of bacterial metagenomic DNA from the digesta fraction

Lq fraction was centrifuged in a 1:1 v/v Percoll gradient (Sigma-Aldrich). The gradient was formed after 20 min centrifugation at 14.000×g and 4°C. The pellet was suspended in lysis buffer (700 mM NaCl, 50 mM Tris-HCl pH 8.0, 100 mM EDTA, 4% SDS) containing 250mg of 0.1mm sterile zirconia beads (BioSpec Products). The cells were disrupted by vortexing during 30 s at maximum speed. After that, the mixture was incubated for 15 min at 70°C, with gently shaking by hand every 5 min and centrifuged for 5 min at 16.000×g and 4°C. The supernatant was transferred to a fresh tube. 15 μl of Proteinase K (0.2 mg/ml) was added and the mixture was further incubated for 1 h at 37°C. A solution of 10% CTAB in 0.7 M NaCl was immediately added and the reaction was incubated for 10 min at 65°C. Two consecutive extractions with equal volumes of phenol were performed. The phases were separated by 10 min centrifugation at 10.400×g and 4°C. The aqueous phase was removed and transferred to a fresh tube. After two consecutive extractions with chloroform, the DNA was precipitated with 0.6 volumes of isopropanol and 0.1 volumes of 3 M sodium acetate pH 5.2 for 30 min on ice. DNA was recovered by centrifugation, washed on 70% ethanol, air dried and re-suspended in water with 20 μl of RNAse A (0.4 mg/ml). The purified DNA was resolved on a 0.8% agarose gel in TAE buffer. Fragments with molecular weight higher than 20 kb were excised from the gel and recovered from the agarose matrix using the QIAEX II Gel Extraction Kit (Qiagen).

### Library construction and screening for lipolytic clones

The metagenomic DNA library was constructed using the CopyControl Fosmid Library Production kit with the pCC1FOSVector (Epicentre) according to the manufacturer´s instructions. MaxPlaxLambda Packaging Extracts (Epicentre) were used for packaging and infection of *E*. *coli* EPI300-T1R (Epicentre), the library host. Transformants were selected by growing in Luria Bertani (LB) [28] agar medium supplemented with 12.5 μg/ml chloramphenicol (LB-Cm) at 37°C for 16 h. The library was arranged in 96-wells microtiter plates with LB-Cm liquid medium. After overnight growth at 37°C, 25% (v/v) glycerol was added and the cells were stored at -20°C. For lipolytic activity screening, clones were replica plated with a 48-pin array onto LB-Cm agar medium containing 1% (v/v) tributyrin (Sigma-Aldrich), 12.5 μg/ml chloramphenicol and 0.01% (w/v) L-arabinose to increase the fosmid copy number. Cells were grown at 30°C and periodically checked for enzymatic activity.

Clones expressing lipolytic activities were identified by the formation of clear halos surrounding the colonies after 2 to 3 days. To confirm that the lipolytic activity was due to the presence of the fosmid, plasmid DNA was isolated from the positive clones and electroporated into fresh cells. The clones that did not replicate the observed phenotype on tributyrin media were discarded. Tributyrin-positive clones were also tested on 1% tricaprylin (Sigma-Aldrich) and 1% triolein (Sigma-Aldrich), which have longer fatty acid chains: C8 and C18, respectively.

### *In vitro* transposon mutagenesis and DNA sequencing

Identification of the open reading frames (ORFs) responsible for the observed lipolytic activities was done by *in vitro* transposon mutagenesis using the EZ-Tn5 <KAN-2˃ Insertion Kit (Epicentre) according to the manufacturer’s instructions. The transformants were screened on solid tributyrin media, as described above, containing 50 μg/mL kanamycin instead of chloramphenicol. Loss-of-function mutants were analyzed for single insertion of the transposon by restriction analysis as follows. Both wild-type and mutant fosmids were digested with BamHI and XhoI. After comparing the fragments sizes between them, single insertion mutants were selected because only one of the fragments from the wild-type was split into two smaller fragments. Flanking DNA was sequenced by conventional Sanger method (Macrogen). ORFs were called using getORF from the EMBOSS suite.

### Est10 cloning

Est10 coding sequence was amplified by PCR with primers 5'-AAAAA**CATATG**ATCATGAAAAAACAGAATTTCTTCG-3' containing a NdeI site shown in bold, and 5'-ATTA**GGATCC**AATCAGTTCTCCATACGG-3' containing a BamHI site shown in bold. PCR was performed using high fidelity Pfu DNA polymerase (recombinant) (Fermentas) according to the manufacturer recommendations. The reactions were done using the following conditions: an initial step of 5 min at 94°C, followed by 30 cycles of 95°C for 30 s, 50°C for 30 s and 72°C for 110 s. The final extension was at 72°C for 5 min. PCR products were resolved on 1% (w/v) agarose gel and fragments with expected product size of 1,100 bp were purified from the gel. Est10 coding sequence was ligated into expression vector pET14b after digestion with NdeI and BamHI, generating an N-terminal 6xHis-tag fusion. The ligation reaction was electroporated into *E*.*coli* DH5α cells and the absence of unintended mutations on the resulting pET14b-Est10 construct was verified by sequencing.

### Overexpression and purification of Est10

*E*.*coli* BL21 (DE3) pLysS cells harboring pET14b-Est10 were inoculated into 1L of 2X YT media (tryptone 16 g/l, yeast extract 10 g/l, NaCl 5 g/l, pH 7.0). When cells reached OD_620_ 0.5–0.7, expression was induced with 1 mM IPTG (isopropyl β-D-1-thiogalactopyranoside) for 18 h at 20°C with shaking. After induction, the culture was stored on ice for 15 min and centrifuged at 1600×g for 30 min at 4°C. Cells were suspended in 15 ml of binding buffer (50 mM imidazole, 300 mM NaCl, 50 mM NaH_2_PO_4_ pH 8.0) and sonicated in an Ultrasonic Homogenizer (Cole-Palmer Instrument. Co) during 6 pulses of 1 min each at 50% duty cycle. Extracts were clarified by centrifugation at 12,000× *g* for 30 min. One ml of 50% Ni-NTA agarose resin (Invitrogen) was used to purify the histidine tagged Est10 from 4 ml of clarified cell extract. Resin bound Est10 was washed with increasing concentrations of imidazole (50–150 mM) in 300 mM NaCl and 50 mM NaH_2_PO_4_ pH 8.0 and eluted with 250 mM imidazole on the same buffer. The eluted protein was then dialyzed twice against the same buffer without imidazole and with the addition of 10% (v/v) glycerol. The purity of the protein was tested on a sodium dodecyl sulfate-polyacrylamide gel electrophoresis (SDS-PAGE). The protein concentration was determined by the bicinchoninic acid (BCA) method using bovine serum albumin (BSA) (Sigma) as a standard.

### Determination of preferred chain length

Six *p*-nitrophenyl (pNP) esters of fatty acids with different chain lengths were obtained from Sigma-Aldrich: pNP acetate (C2), pNP butyrate (C4), pNP decanoate (C10), pNP dodecanoate (C12), pNP myristate (C14) and pNP palmitate (C16). Each enzymatic reaction contained 100 mM sodium phosphate buffer pH 8.0, 4 mg/ml Triton, 0.8 mM of each pNP ester dissolved in acetonitrile:isopropanol mix (80:20 v/v) and 50 nM of purified Est10.

One unit of enzyme activity (U) was defined as the amount of enzyme required to release 1 μmol of *p*-nitrophenol per minute. The production of *p*-nitrophenol was continuously monitored at 405 nm in a Varioskan Flash (Thermo Scientific) during 15 min at 40°C. The activity of the enzyme was calculated by measuring the initial reaction rate. The data were collected in triplicates and a blank reaction without enzyme was included for each substrate.

### The effect of temperature on activity and thermostability

To investigate the effect of temperature on enzymatic activity, enzymatic assays were performed in 100 mM sodium phosphate buffer pH 8.0, 0.3% Triton, 2 mM *p*-NP butyrate dissolved in acetonitrile:isopropanol (80:20 v/v) and 50 nM of Est10. The production of *p*-nitrophenol was continuously monitored at 405 nm in a Varioskan Flash for 15 min at different temperatures (30°C, 35°C, 40°C, 45°C, 50°C and 55°C).

Thermostability was determined by pre-incubating Est10 for 30 min at various temperatures (30°C, 37°C, 40°C, 45°C, 55°C and 65°C) without substrate. The pre-incubated enzyme was then added to the described reaction mix and the reaction was allowed to proceed for 15 min at 40°C. For both assays initial reaction rates were measured from independent triplicate experiments and blank reactions without enzyme were included.

### Effect of pH, cations, chelating agents and detergents on activity

To determine the effect of pH on Est10 activity, the enzymatic hydrolysis was performed using a buffer mix of 25 mM acetic acid, 25mM MES and 50mM TRIS and pH adjusted between pH 3.6–9.5 [29]. The reactions were carried out in this buffer mix, 0.3% Triton X-100, 50 nM Est10 and 2 mM *p*-NP butyrate during 15 min at 40°C.

To test the effects of metal ions, detergents, inhibitors and chelating agents on the activity of the esterase, the enzyme was incubated in their presence at a final concentration of 1 mM of each one for 15 min at room temperature in a reaction mix containing 100 mM sodium phosphate buffer pH 8.0, 0.3% Triton X-100, 50 nM Est10 and 2 mM *p*-NP butyrate. Then the reaction was incubated at 40°C for 15 min. The following additives were analyzed: salts (NiCl_2_, CaCl_2_, Cu_2_SO_4_, MnCl_2_, FeCl_3_·6H_2_O, Cd(CH_3_CO_2_)_2_, ZnSO_4_, CoCl_2_·6H_2_O, MgCl_2_, AgNO_3_), chelating agents (EDTA, EDDHA) or detergents (Tween20, Tween40, Tween60, SDS, CTAB) and other additives such as PMSF, a serine hydrolases inhibitor, and DTT, a reducing agent. Data were collected in triplicate by measuring the absorbance at 405 nm. A blank reaction without enzyme was included.

### Substrate selectivity using an ester library

To determine the substrate selectivity of Est10, we used a library containing the following esters (all provided by Sigma-Aldrich): ethyl acetate, ethyl butyrate, ethyl hexanoate, ethyl octanoate, ethyl decanoate, vinyl acetate, propyl acetate, butyl acetate, phenyl acetate, isopropyl acetate and methyl phenyl acetate. *p*-nitrophenol was used as a pH indicator to monitor ester hydrolysis colorimetrically as previously described [30]. The reactions were carried out with 1 mM of each substrate dissolved in 1% (v/v) acetonitrile, 10 μg Est10 and 0.44 mM *p*-nitrophenol on 1 mM sodium phosphate (pH 7.0). The reactions were monitored by measuring the initial rate of decrease in absorbance at 405 nm during 1 hour at 40°C. Experiments were done in triplicates and a blank reaction without enzyme was included for each substrate.

### Phylogenetic analysis

BlastP [31] was used for homology search among selected protein sequences. Reciprocal score values were used as input for clustering analysis, which was done using the Ward method with Euclidean distance implemented in R programming language. Sequences that belong to Est10 cluster, family XV members (see below), were subsequently analyzed. Protein sequences were aligned using different methods: E-INS-I strategy implemented in MAFFT [32], MUSCLE [33], CLUSTALW2 [34] and PROBCONS [35] programs. Phylogenetic trees were inferred using the Maximum likelihood method by means of PHYML version 3.1 [36]. The default SH-like test was used to evaluate branch supports as recommended by Anisimova et al. [37]. ModelGenerator version 0.85 [38] was used to find the most appropriate model of evolution of amino acid sequences.

### Three dimensional modeling of Est10

Three dimensional (3D) homology modeling was performed by RaptorX Web Server [39] and SWISS-MODEL [40]. The catalytic domain model was built using the 3D structures 1K8Q chain ‘A’ [41] and 1HLG chain ‘A’ [42] as templates. The quality of protein structural models and estimated model error for the predicted position of each residue were estimated by the same servers. Protein structure models were visualized with VMDv1.9.1 [43] available at http://www.ks.uiuc.edu/Research/vmd/ and with JavaScript protein viewer PV from SWISS-MODEL.

### Nucleotide sequence accession number

The DNA sequence of est10 is available in the GenBank database under the accession number KM042178.

## Results

### Metagenomic library construction and screening

To identify genes associated with lipolytic activity, a metagenomic library was generated using DNA isolated from the non-associated bacteria present in the Lq or liquid fraction of cow rumen. The library contained 27.500 clones with average insert size of 42 kbp. The quality and size of inserts were verified by analyzing 40 randomly picked clones. The majority of analyzed clones contained inserts of approximately 35–45 kbp. Restriction analysis revealed a high level of diversity among the cloned DNA fragments (data not shown).

Fosmid clones encoding esterase activity were identified by their halo-forming ability on agar plates containing tributyrin. A total of 3 clones were identified in these plates. None of them showed similar activities in tricaprylin or triolein plates, suggesting that the encoded enzymes are not lipases.

### Identification of lipolytic genes

**Fig 1:**
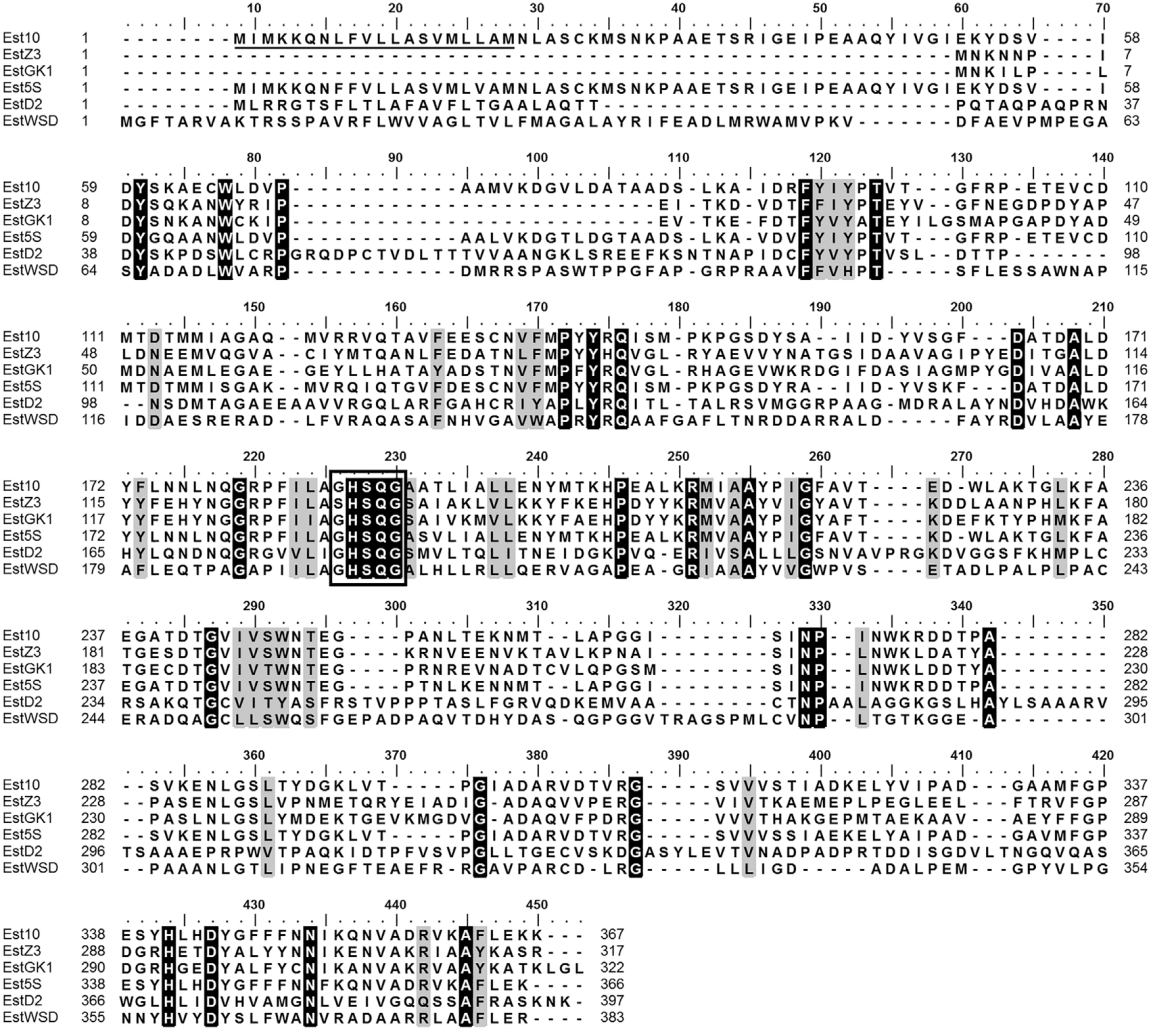
Sequence alignment of Est10 with its major closest homologs. The amino acid sequences correspond to Est10 (GI accession number KM042178), EstZ3 (ADE28720.1, 41% identity with Est10), EstGK1 (ADE28720.1, 39% identity), Est5S (ABI17943.1, 92% identity), EstD2 (ADN26553, 29% identity), and EstWSD (AFY63009, 29% identity). The conserved pentapeptide is shown with a rectangle. Residues that are 100% conserved are shadowed in black, and those between 75% and 100% are shadowed grey. The residues that encompass the putative signal peptide on Est10 are marked with a black line.

*In vitro* transposon mutagenesis was used to identify the genes responsible for the observed lipolytic activity from each fosmid. Loss-of-function mutants were isolated and the single insertion of the transposon was verified by restriction analysis. The flanking regions were sequenced from the transposon arms. The insertion site was mapped inside an ORF of 367 amino acids. The putative gene, designated *est10*, was used to query the non-redundant GenBank protein database using BlastP [31]. Est10 ORF shared 92% sequence identity with Est5S, an esterase from an uncultured bacteria previously found in cow rumen [20]. Est5S was proposed as a member of the novel family XV [17]. Est10 also showed similarity with two other esterases: EstGK1 and EstZ3, 39% and 41% of identity, respectively, isolated from a metagenomic library of sheep rumen [15]. Other related esterases were EstD2 (29% identity) [7], and EstWSD (29% identity) [16] both isolated from soil metagenomes. Interestingly, all of them come from unidentified bacteria (Fig 1).

### Sequence analysis of Est10

The Est10 amino acid sequence contains the pentapeptide GHSQG (Fig 1), which corresponds to the conserved GXSXG motif found on most bacterial and eukaryotic serine hydrolases, such as lipases, esterases, serine proteinases, and β-lactamases [3]. Est10 exhibited the conserved domain DUF3089 and also the common α/β hydrolase domain [11, 44].

A putative signal peptide on Est10 was detected using SignalP [45], and it appears to encompass the first 20 residues (Fig 1). The putative excision site was predicted between residues 33 and 34 (data not showed). This suggests that Est10 can potentially be secreted into the extracellular space explaining the presence of a halo of hydrolysis around the colony on solid media containing tributyrin.

### Phylogenetic analysis of Est10

**Fig 2:**
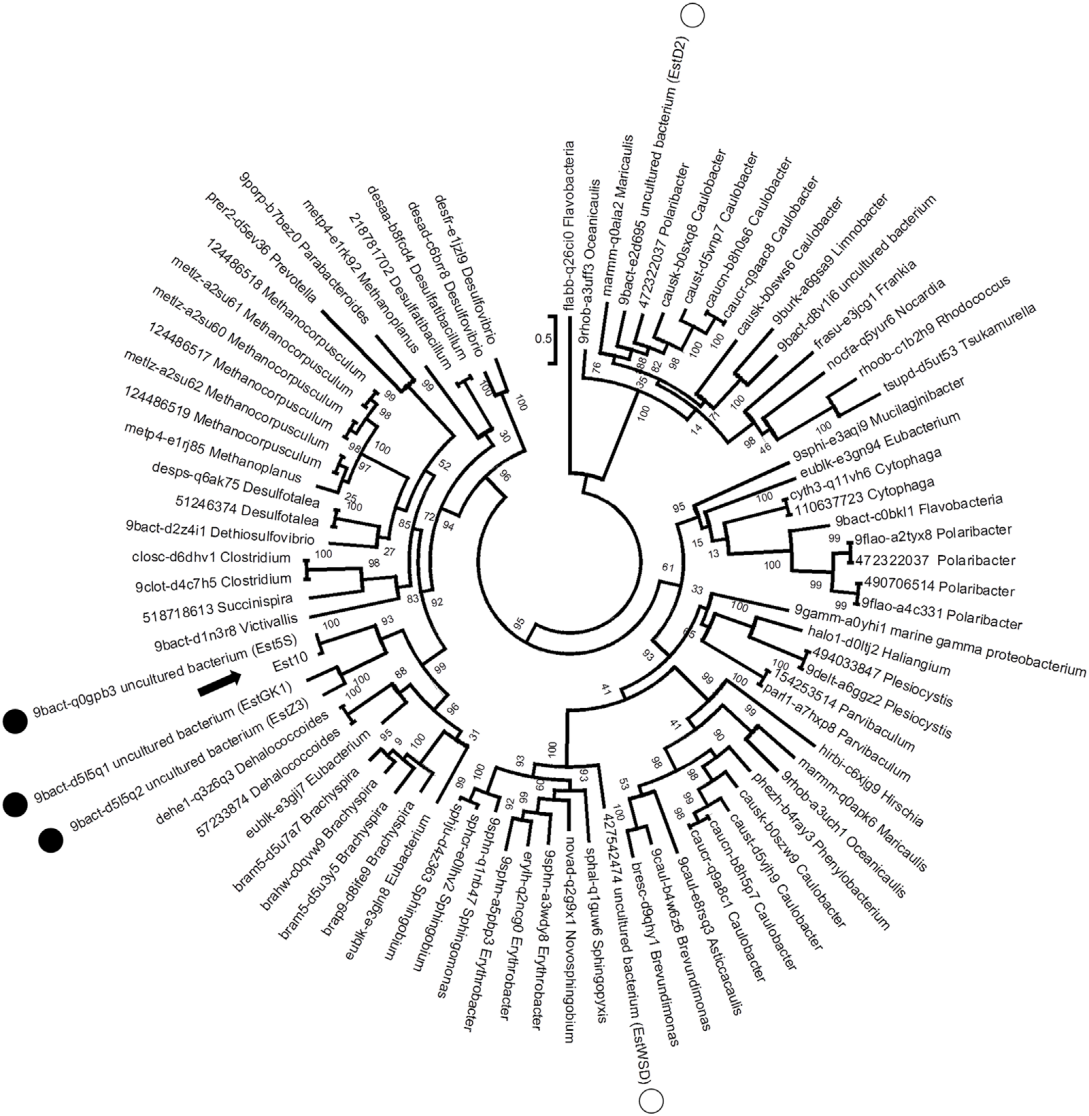
Maximum Likelihood inference of the phylogenetic relationships between members of family XV based on amino acid sequences. Alignment was obtained with MAFFT with the E-INS-I strategy [52]. The numbers of interior branches represent estimated SH-like support values. ESTHER database accession numbers of the sequences used are included in the tree next to the genus of the organisms of origin. GI accession number is included when the protein sequence was retrieved from GenBank database. The position of Est10 is indicated with a black arrow, characterized members of family XV with black circles, and characterized members of the domain family DUF3089 with white circles.

BlastP results and clustering analysis suggested that the homology between protein members of family XV and other esterases is low (S1 Fig). Two main clusters were identified using the classification of bacterial lipolytic enzymes [12], which is based on comparison of amino acid sequences. One of them is exclusively composed of esterases from family XV. Est10 also clustered with esterases from family XV, such as EstD2 and EstWSD. We also analyzed the phylogenetic distribution of the hydrolases comprising the family XV in order to properly place Est10 within it (Fig 2 and S2 Fig). Cladograms based on different alignments showed almost identical results (data not shown). The Maximum likelihood tree showed that Est10 is closely related to Est5S, EstGK1 and EstZ3 with very high statistical support.

### Determination of substrate specificity, effect of pH, temperature and thermostability on Est10 activity

**Fig 3:**
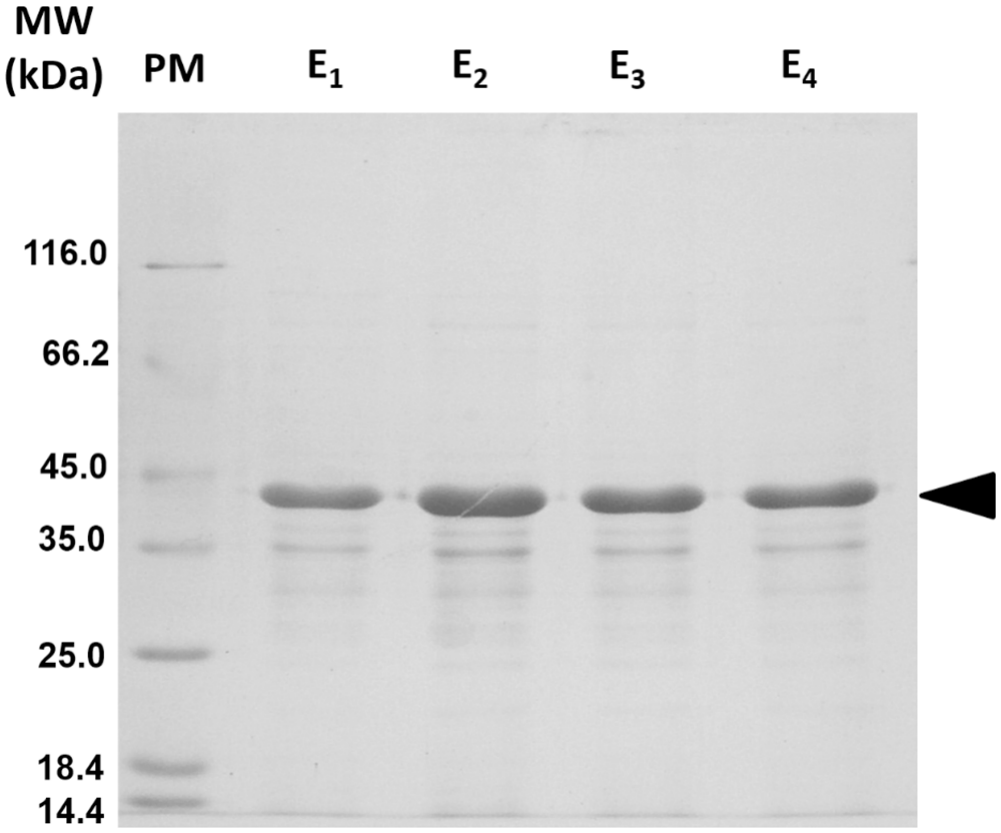
SDS-PAGE analysis of purified 6xHis-Est10 protein stained with Coomassie blue. Recombinant 6xHis-Est10 was purified by affinity chromatography on a Ni^2+^-NTA matrix. Lane PM: protein molecular weight marker. Lanes E1-4: consecutive eluted fractions with 250 mM of imidazol. The position of recombinant Est10 is indicated by a black arrowhead. The calculated molecular weight of Est10 is 40.2 kDa.

**Fig 4:**
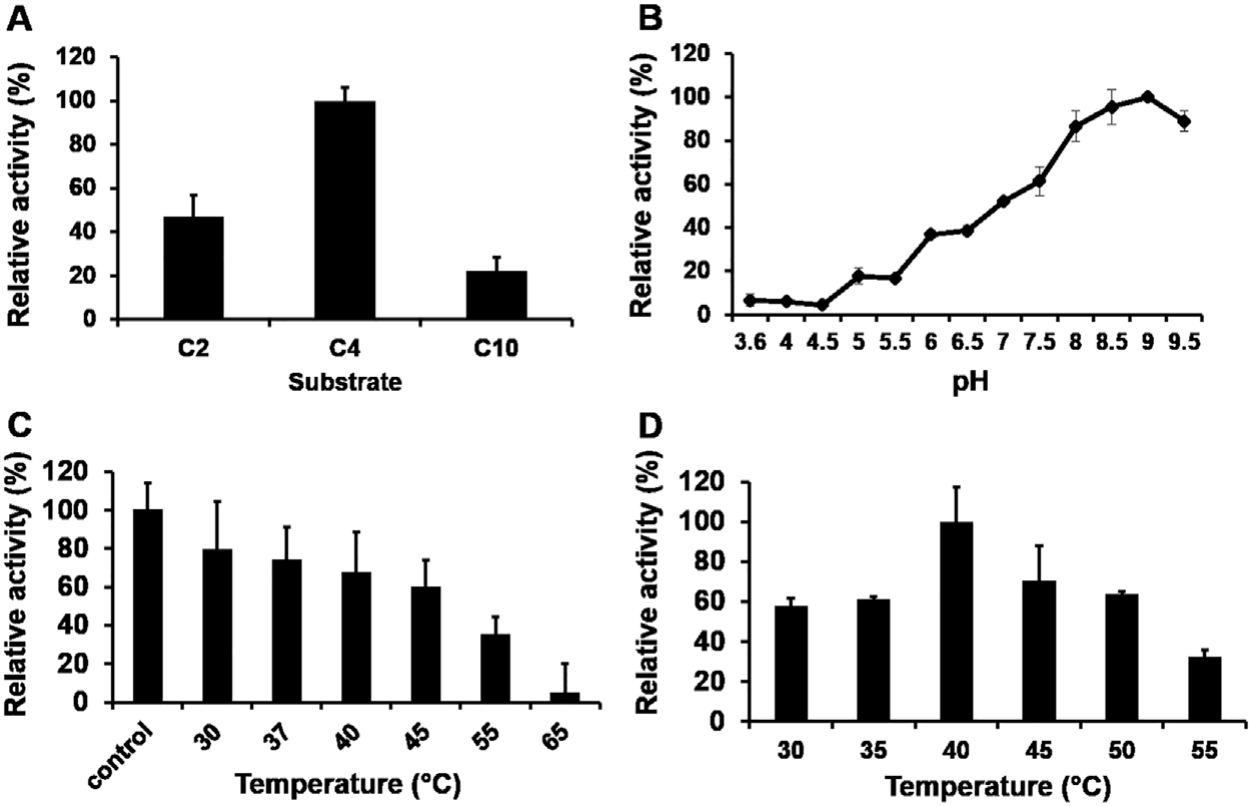
Characterization of Est10 esterase activity. (A) Determination of chain length specificity using pNP esters of fatty acids: acetate (C2), butyrate (C4), and decanoate (C10. (B) Effect of the pH on esterase activity of Est10. (C) Thermal stability of Est10. Est10 was pre-incubated for 30 min at temperatures ranging from 30°C to 65°C before determining its residual activity. The control was not pre-incubated. (D) Effect of the temperature on the esterase activity of Est10. The reaction was carried at temperatures ranging from 30°C to 55°C. Except when noted, reactions were performed at 40°C using pNP butyrate as substrate. In all cases averages of triplicate assays are shown and error bars represent standard deviation.

Est10 was expressed as a His-tagged fusion protein and purified by affinity chromatography using a Ni^2+^ NTA resin (Fig 3). Despite Est10 having the potential to be secreted from its original host, the His-tagged Est10 produced in *E*.*coli* was not detected in the extracellular liquid media. Substrate specificity of the purified enzyme was initially assayed using fatty acids esters of *p*-nitrophenol (Fig 4A). Est10 showed maximum activity towards pN butyrate (C4). Activity against pNP dodecanoate (C12), pNP myristate (C14) and pNP palmitate (C16) was not detected (data not shown). These results are in agreement with the observation that only short chain substrates, like tributyrin, were hydrolyzed by the original Est10 containing clone, and confirm that Est10 is an esterase and not a lipase. The activity of Est10 was tested under buffered conditions over the pH range 3.6 to 9.5, using pNP butyrate (C4) as substrate, at 40°C. Est10 displayed highest levels of activity at pH 9.0 which is uncommon for an esterase (Fig 4B). In order to test its thermostability the enzyme was pre-incubated at various temperatures between 30°C and 65°C for 30 min and its residual activity was assayed. The enzyme activity remained above 60% up to 45°C (Fig 4C). Pre-incubation at higher temperatures resulted in inactivation of the enzyme. The effect of the reaction temperature on the activity of Est10 was determined between 30°C and 55°C, using pNP butyrate as substrate. The maximal observed activity for Est10 was at 40°C, but relatively high activities were observed within the 30°C–50°C temperature range (Fig 4D).

### Effects of cations, detergents, chelating agents and additives on Est10 activity

**Fig 5:**
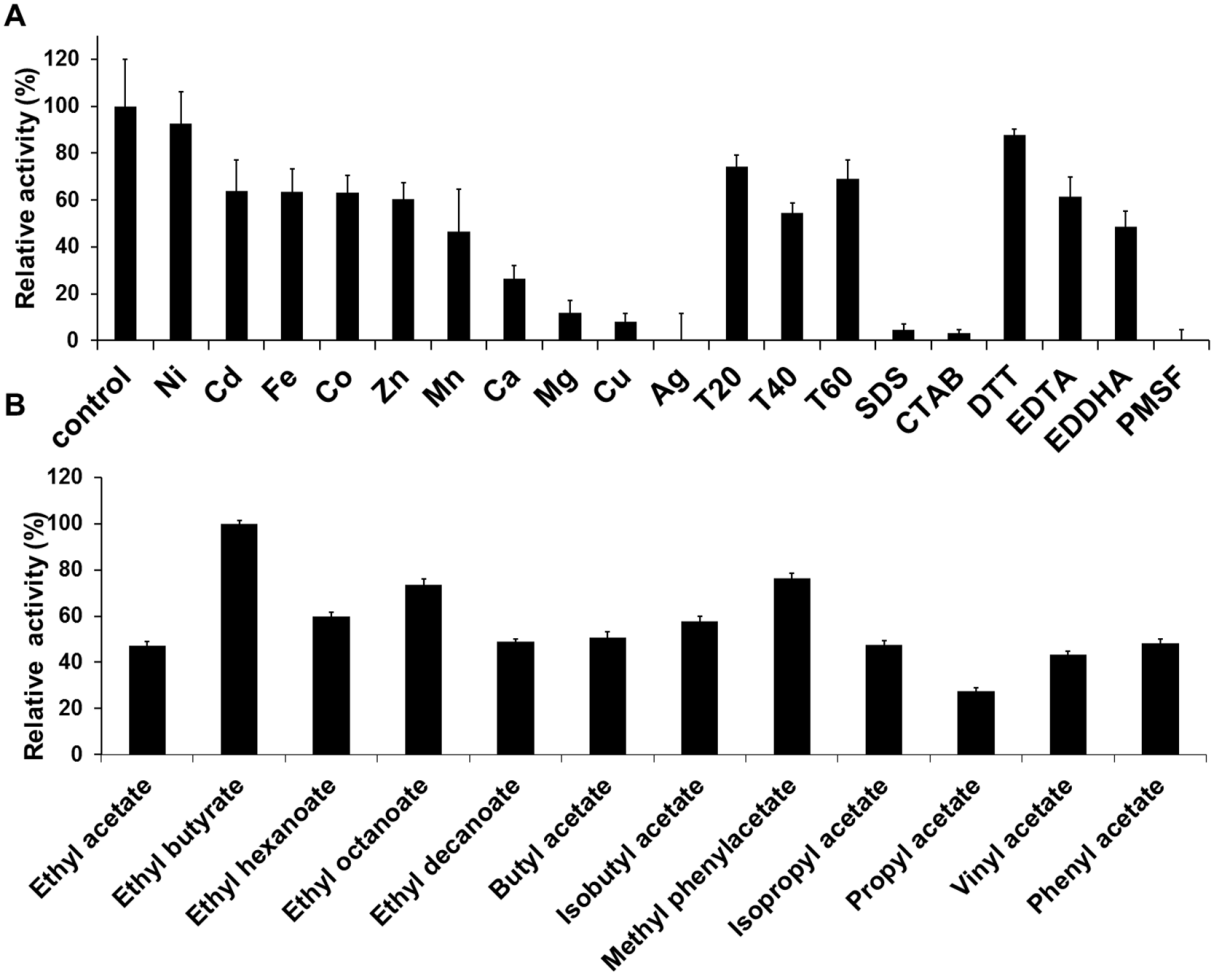
Determination of Est10 substrate specificity and tolerance to metals, detergents, chelating agents and additives. (A) Salts, detergents, chelating agents, and additives were added at a concentration of 1mM to a reaction mix containing 50nM Est10 and 1mM pNP butyrate. Reactions were carried out at 40°C during 15 min. (B) Est10 activity was determined with different esters as substrates. In all cases averages of triplicate assays are shown and error bars represent standard deviation.

Est10 showed various degrees of inhibition by metal ions (Fig 5A). The enzymatic activity was not significantly affected by NiCl_2_, was only partially inhibited by Cd(CH_3_CO_2_)_2_, FeCl_3_, CoCl_2_, ZnSO_4_, and MnCl_2_. However, it was greatly inhibited by CaCl_2_, MgCl_2_, Cu_2_SO_4_, and AgNO_3_. Nonionic detergents Tween20, Tween40 and Tween60 had little effect on Est10, while ionic detergents SDS and CTAB were strong inhibitors. The reducing agent DTT had no effect on Est10 activity while the serine hydrolases inhibitor PMSF completely abolished it. Chelating agents EDTA and EDDHA only partially affected the enzymatic activity.

### Substrate specificity using an ester library

A library of esters was used to evaluate the affinity of Est10 towards different chemical structures present on the ligand. We included ethyl esters of fatty acids of diverse chain lengths as well as esters of alcohol substituents with different geometries. Est10 elicited detectable activities against all of them (Fig 5B). As previously observed, Est10 preferred shorter chains on the fatty acid. The highest activity was obtained with ethyl butyrate (C4) followed by ethyl octanoate (C8). The enzyme retained 76% of its activity when assayed against methyl phenylacetate which is a larger aryl ester. We also tested different alcohol substituents but the esterase activity did not appear to be significantly affected by them.

### Determination of kinetic parameters

The kinetic parameters of the purified Est10 were determined by measuring the hydrolysis of pNP acetate (C2), pNP butyrate (C4) and pNP decanoate (C10) (Table 1). Kinetics parameters were obtained by non-linear least squares implemented on the R package [46]. Est10 achieves saturation at low concentrations of substrate which is characteristic for carboxylesterases. As expected, Est10 elicited maximal specificity constant (k_cat_/K_M_) with pNP butyrate (C4).

**Table 1:** Kinetic parameters of Est10.

Substrate	Specific activity(U/mg of protein)^a^	K_M_(mM)^a^	k_cat_ (s^-1^)^a^	k_cat_/K_M_(s^-1^ mM^-1^)^a^
C2	0.31 (0.04)	0.3 (0.1)	0.22 (0.03)	0.8 (0.4)
C4	4.4 (0.2)	0.16 (0.02)	3.1 (0.1)	19 (3)
C10	1.06 (0.06)	0.35 (0.06)	0.72 (0.04)	2.1 (0.5)

^a^Standard errors are indicated in parentheses.

## Discussion

Enzymes are remarkable biocatalysts, accelerating the rates of a wide range of biochemical reactions and providing solutions for a variety of biotechnological applications. Metagenomic approaches provide access to the total microbiome of different environmental samples and have been successfully employed in mining novel enzymes [26, 47, 48]. In this study, a metagenomic library constructed from bovine rumen was screened for lipolytic genes by function-driven analysis. Unlike an approach based on sequence information, activity-based approaches allow the detection of genes of interest without any presumption about their sequences [23]. Using this approach we were able to identify an esterase, named Est10, from the bacteria inhabiting the liquid fraction of bovine rumen. The organism of origin was not identified and it is likely to be uncultivable.

Est10 is predicted to be a secreted lipolytic enzyme. It was initially identified because of the halo of hydrolysis formed around the colonies on trybutirin solid media. However, the His-tagged Est10 was not detected in the extracellular liquid media. One alternative to explain this discrepancy is that the presence of the 6xHis tag may interfere with the secretion mechanism of the host. However, such an interference was previously observed only in the presence of Ni^2+^ [49]. Another possibility is that the protein can be secreted from its original host but not when expressed heterologously in *E*.*coli* [50]. If this is the case, the halo observed around the colonies on solid media might correspond to protein leaked from senescent cells. Est10 also displays a conserved domain belonging to the family DUF3089 [19]. This domain has unknown function, but it shows the α/β fold characteristic of hydrolytic enzymes. To confirm the lipolytic nature of Est10, it was expressed and purified as a His-tagged fusion protein in *E*. *coli*. Biochemical assays showed that Est10 displays maximum activity towards C4 aliphatic chains and undetectable activity towards C10 and longer chains (Fig 4A). This preference for short length acyl chains confirmed its classification as an esterase rather than a lipase [12]. Similar results were obtained using ethyl esters of fatty acids (Fig 5B). The active site may not accommodate longer aliphatic chains but is still able to accept aryl esters, such as methyl phenylacetate and phenyl acetate, and maintain a relatively high activity. The size of the alcohol substituent of the ester did not appear to have much influence on the observed activity. For example, vinyl acetate, ethyl acetate, and phenyl acetate displayed similar activities.

The chelating agents EDTA and EDDHA reduced the observed activity by 40% and 50%, respectively (Fig 5A). These results suggest that some metal ions may be needed for optimal activity which is not unexpected for lipolytic enzymes [2]. However, we were unable to identify positive effects with a particular metal. In fact, Est10 activity was quite sensitive to divalent cations. Strong inhibition in the presence of Mg^2+^, Cu^2+^, and Ca^2+^ has been previously observed in other esterases [4]. Regarding its stability in the presence of surfactants, Est10 retained most of its activity in nonionic detergents while being inactivated in the presence of stronger ionic ones.

The closest relative of Est10 is Est5S, an esterase also isolated from an uncultivable bacteria from the bovine rumen [20]. Est10 and Est5S showed similar substrate specificities (Fig 4A) and optimum temperatures (Fig 4D). However, Est10 has a more alkaline optimum pH than Est5S (Fig 4B). Est10 optimum pH is 9.0 and the enzyme retains over 85% of its activity between pH 8 and 9.5. This is a remarkable fact of Est10. Only 13% of the carboxylesterases (EC 3.1.1.1) deposited in the BRENDA database display optimum pH above 9 [51].

Est10 optimum temperature of around 40°C allows its classification as a mesophilic esterase, although it retains up to 60% of its activity at much lower temperatures (Fig 4D). Also, the enzyme is not thermostable at high temperatures (Fig 4C). Taken together, these data suggest that Est10 preferred temperature of action is between 30° and 40°C which also happens to be the temperature of the ruminal fluid.

Est10 obeys a Michaelis-Menten kinetics for the preferred C4 substrate but also for C2 and C10 (Table 1). The K_M_ and turnover values obtained (0.16–0.35 mM and 0.22–3.1 s^-1^, respectively) are common among carboxylesterases according to the BRENDA database. The ratio k_cat_/K_M_ is maximal for pNP butyrate in agreement with the higher activity observed with butyrate esters (Figs 4A and 5B).

Multiple sequence alignment revealed that Est10 and its closest homologs, Est5S, EstZ3 and EstGK1, contained the conserved pentapeptide GxSxG found in most lipolytic enzymes (Fig 1) [12]. Both Est10 and Est5S have about 50 extra residues on their N-terminal ends. This region encompasses putative signal peptides that are not present on EstZ3 and EstGK1 indicating that the formers are more likely to be translocated for example via the Sec machinery or another mechanism. Interestingly, all these four enzymes form a separate cluster, which is exclusively composed of esterases isolated from metagenomic samples comprising a sub-family by themselves. The sister cluster is composed of esterases from very different origins, genus *Dehalococcoides*, *Brachyspira* and *Eubacterium* (Fig 2) but all of them, together with two more distant esterases, EstD2 and EstWSD [7, 16], display the conserved domain DUF3089. This domain is a common trait in members of family XV of α/β hydrolases of the ESTHER database [17]. The phylogenetic distances observed in the maximum likelihood tree suggest that this group is characterized by a high sequence diversity, in agreement with the high diversity of the phyla that these sequences come from. On the basis of phylogenetic analysis, we propose that the family XV should only comprise Est10 together with Est5S, EstZ3 and EstGK1. The remaining proteins with a DUF3089 domain are too diverse to be included in the same family of esterases. For example, EstD2 and EstWSD are distant proteins, both phylogenetically and functionally, and may grant the creation of new families after other similar proteins were characterized.

[2, 4]Using automated modeling tools, we were able to locate the nucleophilic Ser on a short loop between a highly conserved α-helix and a four-stranded parallel β-sheet (S2 Fig). This location is expected for most α/β hydrolases. Unfortunately, even using different templates (data not shown), we were not able to map into 3D-models the remaining putative residues of the catalytic triad, His_341_ and Asp_344_ [15], because of very low target-to-template accuracy scores.

Esterases and lipases are important tools for biotechnological applications. Here we have characterized a new esterase produced by a still unidentified bacterium which displays high level of activity in alkaline media. This work helps to better define a recently proposed family of esterases. A better delimitation of this family may help to accurately annotate a large number of related proteins whose functions are still unknown.

## Supporting Information

S1 Fig Hierarchical cluster analysis of esterases based on the scaled inverted pairwise BlastP score. Squared Euclidean distance and the Ward’s method were used for this analysis. The position of Est10 is indicated with a black arrow, while family XV cluster is indicated by dashed lines. Families are indicated between brackets when previously reported, FB and FJ refers to previously reported esterases unassigned to any family [15] and [53], respectively.
(TIF)

S2 Fig Ribbon diagrams of the three-dimensional modeling of Est10 from residues 85 to 224. Est10 conserved domain, comprising residues 84 to 224, was modeled using the 3D structure of the human gastric lipase (PDB-ID: 1HLG chain ‘A’) as template [42] in SWISS-MODEL [54]. The Global Model Quality Estimation (GMQE) was only 0.15 indicating that only a portion of the model may be trustable. In fact, local QMEAN scores for the GHSQG pentapeptide region were above 0.7 representing a high expected accuracy of the model in this region. Predicted 3D model of Est10 using human gastric lipase (PDB-ID: 1HLG chain ‘A’) as template and the SWISS-MODEL algorithm. The catalytic Ser189 is depicted by sticks and indicated with a black arrow. Regions of α-helices and β-strands are drawn. Colors represent model quality and are assigned using QMEAN scores where blue is highest reliability and red is lowest. The estimated model error for the predicted position of each residue suggested that the highly conserved pentapeptide, GHS_189_QG, is modeled with little error. Models were visualized using the JavaScript protein viewer PV.
(TIF)
