# Supplementary figures and images for: Est10: A Novel Alkaline Esterase Isolated from Bovine Rumen Belonging to the New Family XV of Lipolytic Enzymes
Source: PLoS One. 2015 May 14;10(5):e0126651. doi: 10.1371/journal.pone.0126651 (PMC4431682; doi:10.1371/journal.pone.0126651)

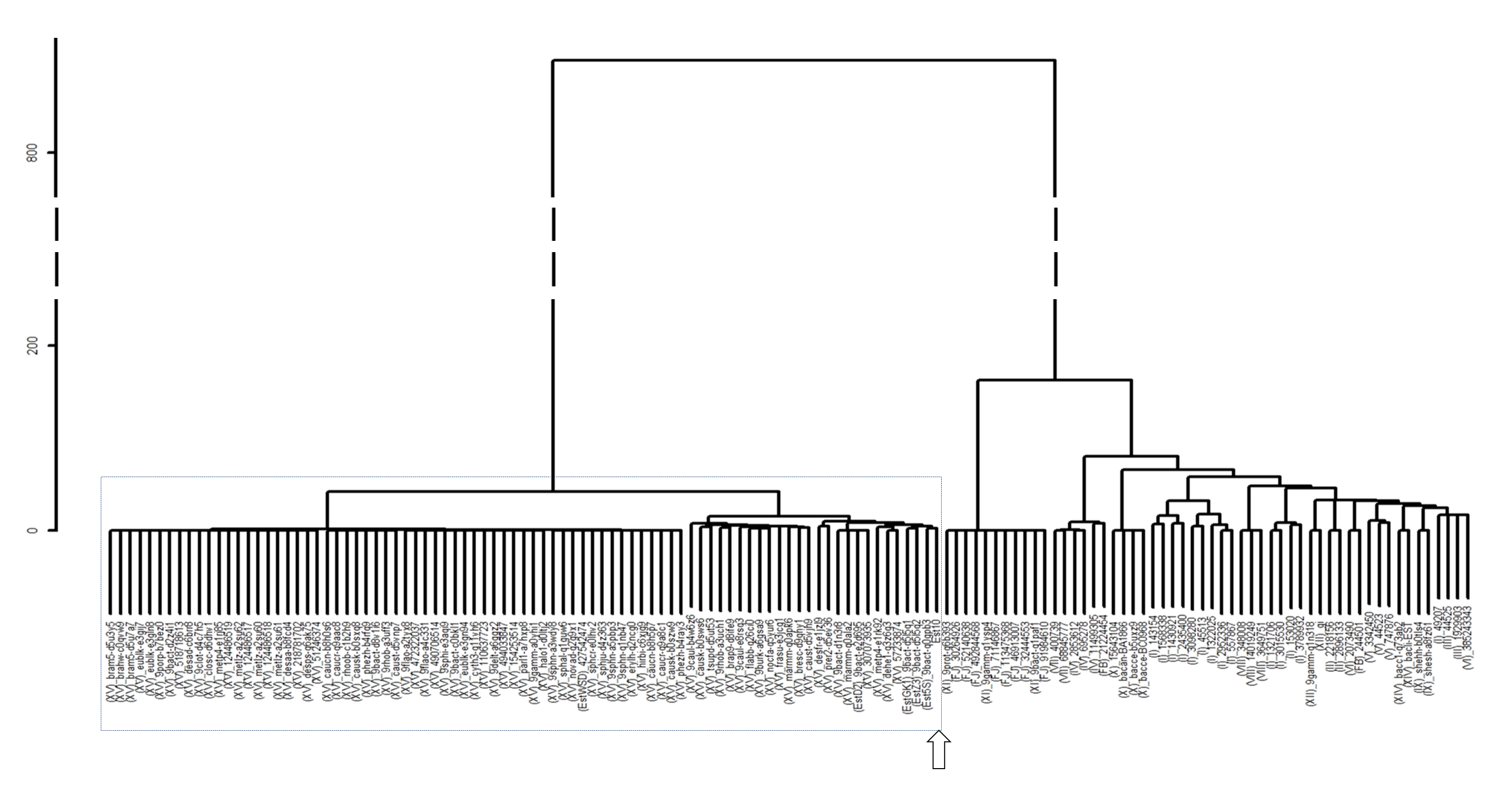

Supplement: S1 Fig — Squared Euclidean distance and the Ward’s method were used for this analysis. The position of Est10 is indicated with a black arrow, while family XV cluster is indicated by dashed lines. Families are indicated between brackets when previously reported, FB and FJ refers to previously reported esterases unassigned to any family [15] and [53], respectively. (TIF) [file pone.0126651.s001.tif]

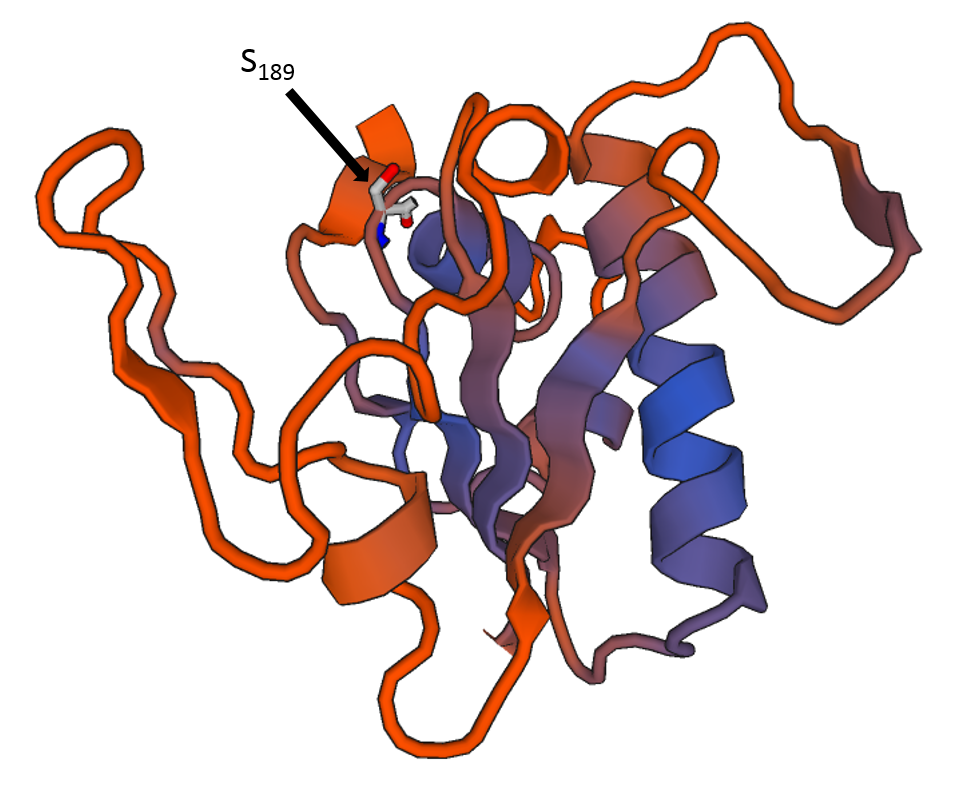

Supplement: S2 Fig — Est10 conserved domain, comprising residues 84 to 224, was modeled using the 3D structure of the human gastric lipase (PDB-ID: 1HLG chain ‘A’) as template [42] in SWISS-MODEL [54]. The Global Model Quality Estimation (GMQE) was only 0.15 indicating that only a portion of the model may be trustable. In fact, local QMEAN scores for the GHSQG pentapeptide region were above 0.7 representing a high expected accuracy of the model in this region. Predicted 3D model of Est10 using human gastric lipase (PDB-ID: 1HLG chain ‘A’) as template and the SWISS-MODEL algorithm. The catalytic Ser189 is depicted by sticks and indicated with a black arrow. Regions of α-helices and β-strands are drawn. Colors represent model quality and are assigned using QMEAN scores where blue is highest reliability and red is lowest. The estimated model error for the predicted position of each residue suggested that the highly conserved pentapeptide, GHS189QG, is modeled with little error. Models were visualized using the JavaScript protein viewer PV. (TIF) [file pone.0126651.s002.tif]
